# Photobiomodulation at Multiple Wavelengths Differentially Modulates Oxidative Stress *In Vitro* and *In Vivo*

**DOI:** 10.1155/2018/6510159

**Published:** 2018-11-11

**Authors:** Katia Rupel, Luisa Zupin, Andrea Colliva, Anselmo Kamada, Augusto Poropat, Giulia Ottaviani, Margherita Gobbo, Lidia Fanfoni, Rossella Gratton, Massimo Santoro, Roberto Di Lenarda, Matteo Biasotto, Serena Zacchigna

**Affiliations:** ^1^Department of Medical, Surgical and Health Sciences, University of Trieste, 34127 Trieste, Italy; ^2^Cardiovascular Biology Laboratory, International Centre for Genetic Engineering and Biotechnology (ICGEB), 34149 Trieste, Italy; ^3^Department of Genetics, Federal University of Pernambuco, Recife, Brazil; ^4^Institute for Maternal and Child Health, IRCCS “Burlo Garofolo”, 34137 Trieste, Italy; ^5^Laboratory of Angiogenesis and Redox Metabolism, Department of Biology, University of Padua, 35131 Padova, Italy

## Abstract

Photobiomodulation (PBM) is emerging as an effective strategy for the management of multiple inflammatory conditions, including oral mucositis (OM) in cancer patients who receive chemotherapy or radiotherapy. Still, the poor understanding of the mechanisms by which the light interacts with biological tissues and the heterogeneity of light sources and protocols employed worldwide significantly limits its applicability. Reactive oxygen species (ROS) are massively generated during the early phases of OM and play a major role in the pathogenesis of inflammation in general. Here, we report the results of a clinical and experimental study, aimed at evaluating the effect of laser light at different wavelengths on oxidative stress *in vivo* in oncologic patients suffering from OM and *in vitro* in two cell types abundantly present within the inflamed oral mucosa, neutrophil polymorphonuclear (PMN) granulocytes, and keratinocytes. In addition to standard ROS detection methods, we exploited a roGFP2-Orp1 genetically encoded sensor, allowing specific, quantitative, and dynamic imaging of redox events in living cells in response to oxidative stress and PBM. We found that the various wavelengths differentially modulate ROS production. In particular, the 660 nm laser light increases ROS production when applied either before or after an oxidative stimulus. In contrast, the 970 nm laser light exerted a moderate antioxidant activity both in the saliva of OM patients and in both cell types. The most marked reduction in the levels of ROS was detected in cells exposed either to the 800 nm laser light or to the combination of the three wavelengths. Overall, our study demonstrates that PBM exerts different effects on the redox state of both PMNs and keratinocytes depending on the used wavelength and prompts the validation of a multiwavelength protocol in the clinical settings.

## 1. Introduction

Chemotherapy (CT) and radiant therapy (RT) are two of the most common and widely used treatments for both solid and haematologic tumors. While advances are constantly made to improve their specificity towards cancer cells in order to limit toxic effects on surrounding tissues, both therapies often lead to debilitating side effects, such as nausea, vomiting, diarrhea, and mucositis/dermatitis. Oral mucositis (OM) is a severe inflammation of the oral and oropharyngeal mucosa, leading to the development of extensive erythema and ulcerations. OM importantly limits the quality of life of patients, as it causes oral pain, dysgeusia, dysphagia, incapacity of autonomous nutrition with the consequent need of parenteral alimentation, and narcotic analgesia. In severe cases, the anticancer therapy has to be either reduced or stopped, with a significant negative impact on overall patient prognosis [1, 2]. The prevalence of OM raises up to 90–100% in patients receiving RT for head-neck cancer malignancies, CT, or high-dose myeloablative chemotherapy for hematopoietic stem cell transplantation [3]. While the etiology and the pathogenic mechanisms of OM have not been fully elucidated yet, several studies have reported a major role of DNA damage and reactive oxygen species (ROS) during the early phases of oral and intestinal mucositis [4–7]. Thus, either limiting ROS generation or increasing their detoxification during CT/RT stands as a rational and promising strategy to prevent OM onset and/or reduce its severity [8–11].

Various approaches have been proposed to manage OM, by either reducing its severity or preventing its onset, including cryotherapy, growth factors, and anti-inflammatory drugs, with very minimal efficacy [12, 13]. Laser therapy, also known as photobiomodulation (PBM), is emerging as an effective intervention in the management of OM [14], and it was recently added to the guidelines issued by both the Mucositis Study Group of the Multinational Association of Supportive Care in Cancer and the International Society of Oral Oncology for the prevention of OM in oncologic patients [15]. Following these guidelines, we have successfully applied PBM to foster the healing of OM lesions in both adult [16–18] and pediatric [19] patients. Yet the exact mechanism by which light at certain wavelengths promotes mucosal healing, also reducing pain and inflammation, is not clearly understood [20, 21].

PBM is based on the assumption that red (600–700 nm) and near-infrared (NIR, 770–1200 nm) light at low irradiance excites specific chromophores, such as the cytochrome c oxidase, essentially located inside the mitochondria, which represents the main source for intracellular ROS generation. *In vitro* studies have so far provided conflicting results on the effect of PBM on ROS balance. While PBM induces a modest and dose-dependent increase in ROS production in normal cell lines, it appears to reduce ROS levels in cells previously exposed to oxidative stress [22, 23]. Whether these effects of PBM can be reproduced on various cell types and to what extent the various wavelengths are differentially able to modulate ROS production still remain open questions.

Here, we report the results of a clinical and experimental study, aimed at evaluating the effect of PBM on oxidative stress *in vivo* in oncologic patients suffering from CT/RT-induced OM and *in vitro* in keratinocytes and neutrophil polymorphonuclear (PMN) granulocytes. In addition, we exploited the roGFP2-Orp1 sensor to monitor redox changes in response to oxidative stress and laser light at different wavelengths in immortalized human keratinocytes.

## 2. Materials and Methods

### 2.1. Laser Device

A gallium arsenide (GaAs) + indium gallium aluminum arsenide phosphide (InGaAlAsP) diode laser device (class IV, K-Laser Cube series, K-Laser d.o.o., Sežana, Slovenia) was used to perform all the experiments. The laser device is associated with a programmable scanner conveniently designed to provide uniform irradiation to different multiwell plates (12, 24, and 96-well plates). Plate covers were removed during irradiation, and the emission tip was held perpendicular above the cells. The emitted light completely covered the irradiated field of each culture plate, and power was adapted to the spot size to provide the desired irradiance and fluence using an optical power meter (LaserPoint Plus+, Via Burona, 51-20090 Vimodrone, Milan, Italy). The device is able to provide 660 nm, 800 nm, and 970 nm wavelength laser light in different combinations of power and energy.

### 2.2. Clinical Study

The study was performed according to the ethical standards of the 1975 Declaration of Helsinki (7th revision, 2013) upon approval by the local ethical committee. All subjects enrolled in the study signed an informed consent to participate. A total of 10 patients affected by OM were enrolled at the Oral Medicine and Pathology Department, Ospedale Maggiore, Trieste, Italy according to the following inclusion criteria:

- Age between 40 and 95 years
- Diagnosis of a solid or haematologic malignancy undergoing CT and/or RT
- Presence of OM of grade 2 or 3 (CTC) related to ongoing oncological therapies
- Availability to undergo PBM for four consecutive days (T0, T1, T2, T3, and one follow-up recall, CTRL)

The exclusion criteria are the following:

- OM previously treated by PBM
- Application of topical oral medication
- Systemic antioxidant therapy

**Figure 1:**
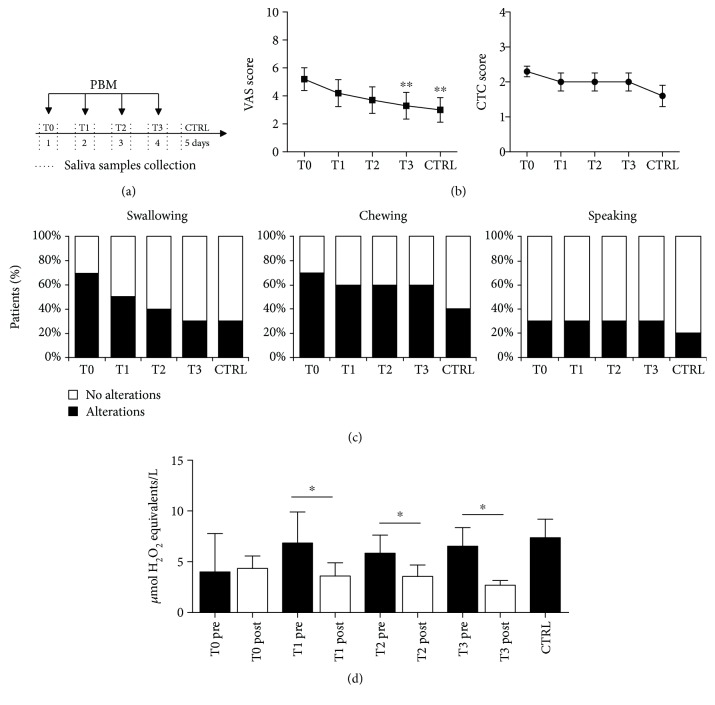
Effect of PBM on clinical parameters and oxidative stress in OM patients. (a) Schematic representation of the study design. Enrolled patients were treated with PBM for 4 consecutive days followed by a control session on day 5. Saliva samples were collected on treatment days before and after each PBM session and once on day 5. (b) Evaluation of OM severity by VAS score (left panel) and CTC score (right panel) over time. Both parameters significantly decreased over time (Friedman's test *p* = 0.0003 for VAS and *p* = 0.0034 for CTC). ^∗∗^Dunn's multiple comparison test *p* < 0.001 compared to T0. (c) Percentage of patients indicating the presence (black bars) or absence (white bars) of either pain or functional alterations in swallowing, chewing, or speaking over time. (d) TOS levels in the patient saliva at the indicated time points. Data are the means ± SD. ^∗^Mann–Whitney *U* test *p* < 0.05.

A schematic representation of the study design is shown in Figure 1(a). During the first visit on day 0 (T0), experienced clinicians registered information about patients' clinical history and scored OM severity according to the common toxicity criteria (CTC) scale (WHO, 1976), considering ulceration and erythema distribution and size. A 0–10 visual analogue scale (VAS) was employed to quantify subjective parameters, including pain, difficulties in swallowing, speaking, and chewing. The patients were treated with PBM at T0, T1, T2, and T3 using a previously optimized protocol (*λ* 970 nm, 200 mW/cm^2^, 6 J/cm^2^, in continuous wave) [18, 24]. Both patients and operators wore protective glasses during the treatment to avoid possible eye damage. PBM was provided using a rotatory motion all over the oral cavity to cover both ulcerated and healthy areas, keeping a 3 cm distance between the laser probe and the tissue. Irradiation time was calculated considering a mean oral mucosa surface area of 215 cm^2^ [25].

Unstimulated saliva samples were collected for 5 minutes before and after each PBM session (T0–T4) and once on day 5 (CTRL). Patients were asked to fast for at least 2 hours before sampling, avoiding tooth brushing, excess alcohol intake, and physical activity since the previous evening. Patients rinsed the mouth with water for 1 minute and then spit saliva for 5 minutes in a sterile collection tube. Samples were stored at −20°C until analysis.

### 2.3. TOS Assay

Oxidative stress status was measured in saliva at each time point using the total oxidant status (TOS) method [26]. This method is based on the principle that oxidant species present in the saliva oxidize the ferrous ion-o-dianisidine complex to ferric ion, creating a coloured complex with xylenol orange in an acidic medium. In each well of a 96 multiwell plate, centrifuged saliva (35 *μ*L) was added to 225 *μ*L of reagent 1 (xylenol orange 150 *μ*M, NaCl 140 mM, and glycerol 1.35 M in 25 mM H_2_SO_4_ solution, pH 1.75). Subsequently, 11 *μ*L of reagent 2 (ferrous ion 5 mM and o-dianisidine 10 mM in 25 mM H_2_SO_4_ solution) was added. The solutions were gently mixed for 5 minutes, and then the absorbance was measured with a multiwell reader spectrophotometer (Glomax multi+ detection system, Promega, Italy) at OD_560_. The absorbance measured before mixing reagent 1 and reagent 2 was used as a sample blank. The assay was calibrated using standard aqueous solutions of hydrogen peroxide (H_2_O_2_). TOS values are reported as the mean of 4 measurements ± standard deviation and are expressed as micromolar hydrogen peroxide equivalent per liter (*μ*mol H_2_O_2_ Equiv/L).

### 2.4. Cells

Human keratinocytes (HaCaT) were maintained in DMEM culture medium supplemented with 10% fetal bovine serum, 100 U/mL penicillin/streptomycin, and 2 mM glutamine (Euroclone, Pero, Milan, Italy). Cells were seeded one day prior to the experiment (10,000 cells/well in 96 multiwell plates and 50,000 cells/well in 24 multiwell plates), avoiding the use of cells over the 10th passage.

### 2.5. Evaluation of Intracellular ROS Production and Kinetics in PMN

PMN isolation from the venous whole blood of 5 healthy volunteers was performed using a Ficoll gradient (Ficoll-Paque Plus, GE Healthcare) through a centrifugation of 400×*g* for 30 minutes. Erythrocytes were lysed with a buffered ammonium chloride solution (ammonium [Tris (1.7 mM)-NH4Cl (16 mM)]). The PMNs were washed in HANKS balanced salt solution (150×*g* for 10 minutes) and seeded at a concentration of 10^6^ cells/mL in RPMI-1640 medium (Sigma-Aldrich) in 96-well plates (100 *μ*L in each well).

After isolation, PMNs were either stimulated with 100 ng/mL LPS (lipopolysaccharide from *Escherichia coli* 055:B5) for 15 minutes or not and then exposed to laser light at either 970 nm (*λ* 970 nm, irradiance 200 mW/cm^2^, fluence 6 J/cm^2^) or 660 nm (*λ* 660 nm, irradiance 50 mW/cm^2^, fluence 3 J/cm^2^). The cells were then incubated for 10′ with 2′7′dichlorofluorescein diacetate (DCFH-DA, Sigma-Aldrich, Saint Louis, Missouri, USA), and OD_529_ emission was measured using a spectrophotometer (Envision plate reader, PerkinElmer, Waltham, Massachusetts, USA) for 2 hours (1 read/minute). ROS levels are reported as the mean of 4 measurements ± standard deviation.

### 2.6. Real-Time Quantification of Oxidative Stress Using Fluorescent Protein-Based Redox Probes in Keratinocytes

HaCaT cells were seeded three days before the experiment in 8-well Ibidi *μ*-slides (100,000 cells/well). The following day, cells were transfected using lipofectamine 2000 with a plasmid expressing roGFP2-Orp1 [27]. After 48 hours, the cells were treated with different PBM protocols [24] and imaged at a Leica LSM 810 confocal microscope.

The following laser protocols were applied, either individually or in combination: (i) *λ* 660 nm, irradiance 50 mW/cm^2^, fluence 3 J/cm^2^, continuous wave; (ii) *λ* 800 nm, irradiance 200 mW/cm^2^, fluence 6 J/cm^2^, continuous wave; and (iii) *λ* 970 nm, irradiance 200 mW/cm^2^, fluence 6 J/cm^2^, continuous wave. An oxidative stimulus (0.5 mM H_2_O_2_) was added either 10 seconds prior to the laser light or immediately after the laser light. Cells were imaged for 240 seconds (12 frames every 20 seconds). The ratio between the fluorescence excited at 408 nm and 488 nm was used as a surrogate indicator of the redox status of the cells, with an expected increase in the 408/488 nm ratio in the presence of oxidative stress. Images were analyzed using the ImageJ software. ROS levels are reported as the mean of 4 measurements ± standard deviation.

### 2.7. Keratinocyte Viability and Oxidative Stress Assay following 5-FU

5-FU 0.1 mg/mL (F6627, Sigma-Aldrich, Saint Louis, Missouri, USA) was added to HaCaT cells for 18 hours, followed by PBM using combined laser wavelengths (*λ* 660 nm, irradiance 50 mW/cm^2^, fluence 3 J/cm^2^; *λ* 800 nm, irradiance 200 mW/cm^2^, fluence 6 J/cm^2^; and *λ* 970 nm, irradiance 200 mW/cm^2^, fluence 6 J/cm^2^). After additional 24 hours, the MTT assay was performed following the manufacturer's instructions (Trevigen, Gaithersburg, Maryland, USA). Results are expressed as % OD_570_ absorbance relative to untreated cells. ROS production was measured 30 minutes after PBM using the cell-permeant 2′,7′-dichlorodihydrofluorescein diacetate (H_2_DCFDA) dye (D399, Invitrogen, Thermo Fisher Scientific, Waltham, Massachusetts, USA), and the obtained fluorescence was normalized on living cells. The results are reported as the mean of 8 measurements ± standard deviation.

### 2.8. Gene Expression Analysis

Total RNA was extracted 30 minutes after PBM using Eurogold Trifast reagent (EMR507100, Euroclone, Pero, Milan, Italy) following the manufacturer's instructions and retrotranscribed using the High-Capacity cDNA Reverse transcription kit (Thermo Fisher Scientific, Waltham, Massachusetts, USA). Taq-manTM probes for HMOX1 (hs00167309_m1) and SOD2 (hs01110250_m1) genes and *β*-actin (as calibrator and reference, ACTB: Hs99999903_m1) were used to quantify the relative mRNAs using an Applied Biosystems 7900HT Fast Real-Time PCR System (Thermo Fisher Scientific, Waltham, Massachusetts, USA) platform. Data were analyzed using the Relative Quantification manager software (Thermo Fisher Scientific, Waltham, Massachusetts, USA) using untreated cells as reference. The experiment was performed in three replicates including duplicate wells, and the results are expressed as the mean ± standard deviation.

### 2.9. Statistical Analysis

The Prism 6.0 software (GraphPad Software, La Jolla, California, USA) was used to perform statistical analysis. All statistical assessments were two-sided, and a *p* value < 0.05 was used for the rejection of the null hypothesis. Friedman's test was employed to test the significance of changes over time in VAS and CTC scores, while Dunn's multiple comparison test was used as post hoc to compare each pair of time points. Pearson's nonparametric correlation test was used to evaluate the relation between TOS levels and VAS or CTC scores at each time point. The Mann–Whitney *U* test was employed to evaluate differences between treated and untreated samples. The Mann–Whitney *U* test was performed to determine gene expression differences among samples. Linear regression analysis was employed to evaluate differences in PMN ROS levels among groups and over time. Two-way ANOVA was applied to determine the significance of differences between the curves representing oxidative status in keratinocytes with genetically encoded redox probes treated with H_2_O_2_ with and without PBM.

## 3. Results

### 3.1. PBM Improves Clinical Parameters in OM Patients and Determines a Transient Reduction in the Oxidant Status of the Saliva

Baseline demographic and clinical characteristics of the 10 patients suffering from OM who completed the study are reported in Table 1. PBM was performed daily for 4 consecutive days (T0 to T3, Figure 1(a)) and well tolerated by all patients, without any adverse event. Effectiveness of PBM was confirmed by the progressive decrease of both the VAS and the CTC scores over time, as reported in Figure 1(b) (Friedman's test *p* = 0.0003 for VAS and *p* = 0.0034 for CTC). The number of patients reporting discomfort in subjective parameters (swallowing, chewing and speaking) also decreased over time following PBM (Figure 1(c)).

In the same patients, we analyzed the total oxidant status (TOS) in the saliva before and after each PBM session, as well as the day following the last session (CTRL). TOS level was significantly correlated to the VAS score at T3 and T4 (Pearson's nonparametric correlation test *p* < 0.05), but not to the CTC score. While the oxidative stress did not change after PBM at T0, starting from T1, ROS levels decreased promptly after each PBM session (Mann–Whitney *U* test *p* < 0.01 for T1, T2, and T3). Despite this constant trend, ROS levels increased again during the following 24 hours, as well as at day 5 (CTRL). Thus, PBM transiently reduces oxidative stress after treatment, but this effect lasts less than 24 hours.

### 3.2. PBM at Different Wavelengths Modulate ROS Production in Both Stimulated and Unstimulated PMNs

**Figure 2:**
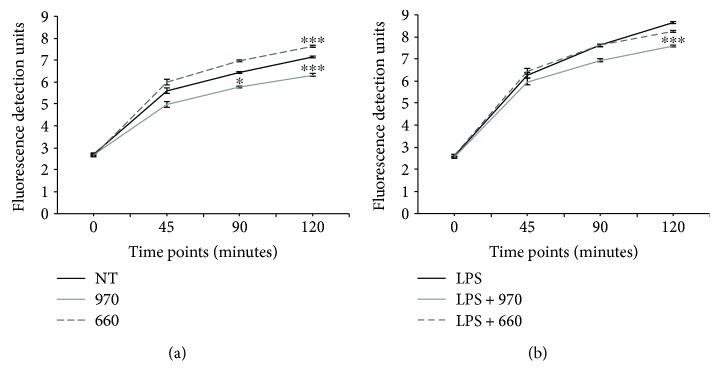
Effect of PBM on intracellular ROS production in unstimulated PMNs and in PMNs stimulated with LPS. (a) Monitoring of fluorescence detection units at OD_529_ over time in unstimulated PMNs. NT: not treated, 970: treated with 970 nm laser light, and 660: treated with 660 nm laser light. ^∗^Linear regression analysis *p* < 0.05 compared to NT. ^∗∗∗^Linear regression analysis *p* < 0.0001 compared to NT. (b) Monitoring of fluorescence detection units at OD_529_ over time in PMNs stimulated with LPS. NT: not treated, 970: treated with 970 nm laser light, and 660: treated with 660 nm laser light. ^∗∗∗^Linear regression analysis *p* < 0.0001 compared to NT.

As PMNs are known to be a major source of ROS in inflammatory conditions, including OM, we investigated the potential of PBM, set at two different wavelengths commonly employed in the clinics (970 nm and 660 nm), to modulate ROS production in unstimulated and LPS-stimulated PMNs. In unstimulated PMNs (Figure 2(a)), the two wavelengths had opposite effects. While the 970 nm protocol significantly reduced ROS levels (linear regression *p* = 0.023 and *p* < 0.0001 after 90 and 120 minutes, respectively), the 660 nm protocol increased ROS levels at 2 hours after PBM (linear regression *p* < 0.0001). As expected, stimulation of PMNs with LPS resulted in increased ROS production in all experimental groups (Figure 2(b)). In line with the results obtained with unstimulated PMNs, we observed a reduction in ROS levels upon irradiation with the 970 nm protocol, whereas the 660 nm did not exert any effect.

### 3.3. Dynamic Real-Time Detection of PBM-Induced Redox Changes in Keratinocytes Using Genetically Encoded Fluorescent Sensors

Compared to standard ROS detection methods, based on the use of chemical indicators, genetically encoded probes, exploiting redox-sensitive fluorescent proteins, allow specific, quantitative, and dynamic imaging of redox events in living cells [27]. To monitor redox changes in keratinocytes upon PBM, we employed a genetically encoded probe based on the redox-active green fluorescent protein 2 (roGFP2) fused to Orp1, a highly sensitive thiol peroxidase that is oxidized by H_2_O_2_ [28]. By introducing the roGFP2-Orp1 probe in keratinocytes, we could monitor any change in the intracellular redox state upon exposure to PBM either in the absence or in the presence of H_2_O_2_-induced oxidative stress. This time we compared the effect of the three wavelengths (660 nm, 800 nm, and 970 nm) that have been so far considered for the treatment of OM and other inflammatory conditions.

**Figure 3:**
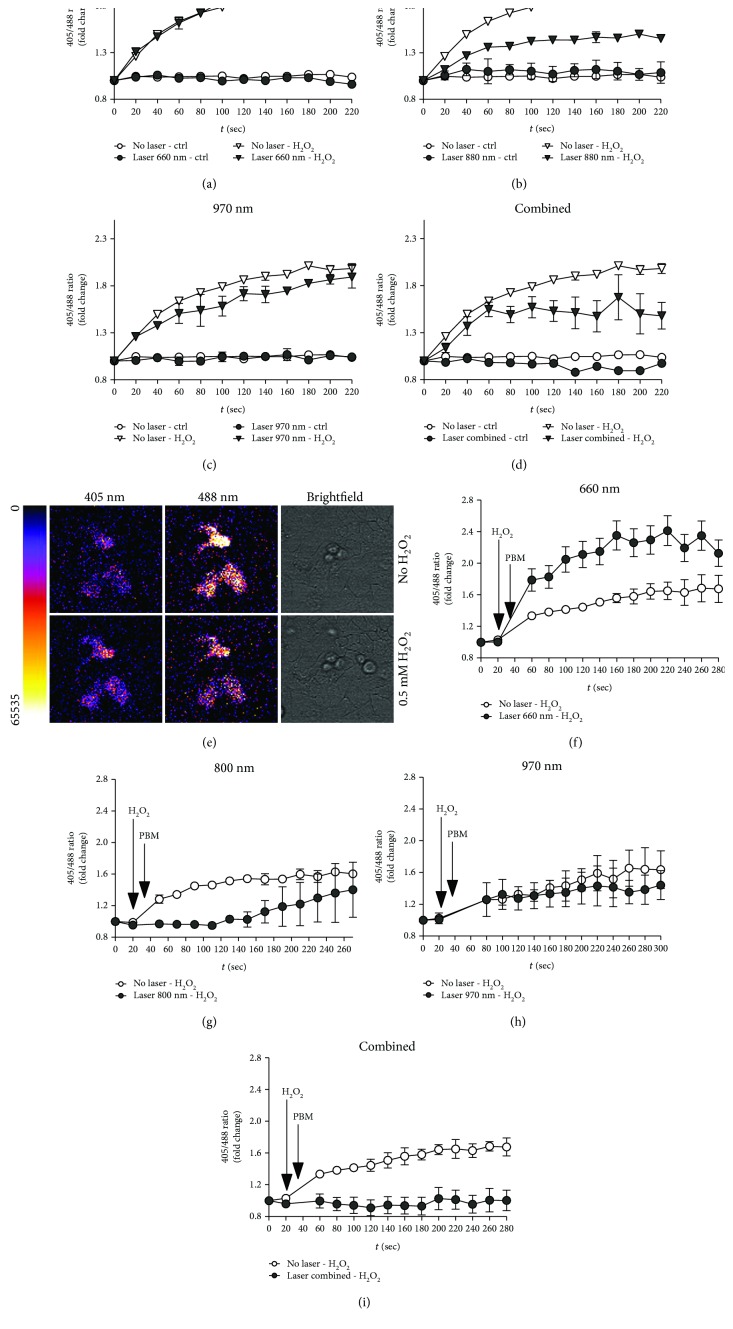
Real-time evaluation of the effect of PBM on the redox status in HaCaT cells using genetically encoded fluorescent sensors. (a–d) Cells were treated with PBM at the indicated wavelength (660 nm in (a), 800 nm in (b), and 970 nm in (c) and the combination of the three wavelengths in (d)) and subsequently exposed to 0.5 mM H_2_O_2_. Measurement of fluorescence started immediately after exposure to oxidative stress. Data are the means ± SD. Signals recorded in treated cells and in cells treated only with PBM are also plotted. (e) Representative images of the fluorescence intensity at 405 nm (left) and 488 nm (center) and transmitted light (right) of the same cells at baseline (upper raw) and upon treatment with 0.5 mM H_2_O_2_ (lower raw). (f–i) Cells were first treated with 0.5 mM H_2_O_2_ and subsequently exposed to PBM at the indicated wavelength (660 nm in (a), 800 nm in (b), and 970 nm in (c) and the combination of the three wavelengths in (d)). Measurement of fluorescence started 20 seconds prior to the exposure to oxidative stress. Data are the means ± SD.

First, we determined whether PBM induces any change in the redox state of cells at baseline and found that any of the three tested laser protocols, and not even their combination, exert a significant effect (Figures 3(a)–3(d)). As expected, the addition of H_2_O_2_ determined a rapid increase in ROS levels (Figure 3(e)). Whereas the 660 nm protocol did not induce any change in ROS production (*p* = ns), the 970 nm protocol and, even more evident, the 800 nm protocol significantly reduced ROS levels (*p* < 0.01 and 0.001, respectively; Figures 3(a)–3(c)). Of notice, the simultaneous delivery of the three wavelengths was also very effective in reducing the oxidative status upon exposure to H_2_O_2_ (*p* < 0.01; Figure 3(d)). We also tested whether PBM was able to modulate ROS levels when applied to cells previously exposed to H_2_O_2_. In agreement with the results obtained in PMNs, the 660 nm protocol further increased ROS levels (*p* < 0.01; Figure 3(f)). In contrast, both the 800 nm and the combined protocols significantly reduced ROS production also in this condition (*p* < 0.01 and *p* < 0.05, respectively; Figures 3(g)–3(i)).

### 3.4. PBM Improves Keratinocyte Resistance to Oxidative Stress Induced by 5-Fluorouracil In Vitro

**Figure 4:**
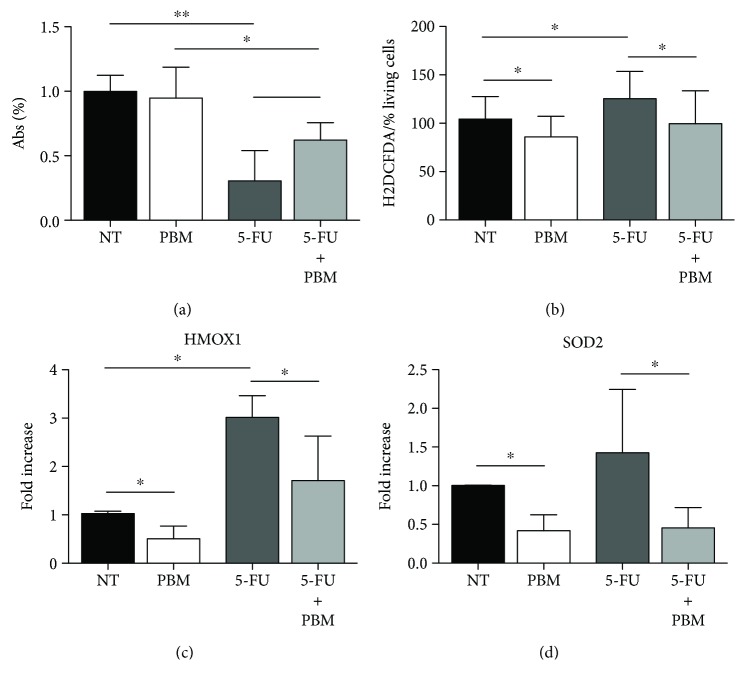
Effect of PBM on keratinocyte survival and oxidative stress induced by exposure to 5-FU (0.1 mg/ml). (a) MTT assay showing the percentage of living cells in the absence of any treatment (not treated, NT) and after exposure to either PBM, 5-FU, or their combination. (b) ROS production (normalized to the percentage of living cells) in the absence of any treatment (not treated, NT) and after exposure to either PBM, 5-FU, or their combination. (c) Levels of HMOX1 gene expression in the absence of any treatment (not treated, NT) and after exposure to either PBM, 5-FU, or their combination. (d) Levels of SOD2 gene expression in the absence of any treatment (not treated, NT) and after exposure to either PBM, 5-FU, or their combination. ^∗^Mann–Whitney *U* test *p* < 0.05; ^∗∗^Mann–Whitney *U* test *p* < 0.01. Data are the means ± SD.

Based on the promising results obtained using the multiwavelength PBM protocol, we wanted to confirm its actual capacity to reduce oxidative stress in keratinocytes treated with 5-fluorouracil (5-FU) treatment, partially mimicking the scenario of OM patients. Indeed, 5-FU is widely used in patients affected by different solid tumors, including five patients enrolled in our study. 5-FU acts as an inducing cell apoptosis through several mechanisms, including the generation of mitochondrial oxidative stress [29]. We treated human keratinocytes with 5-FU for 18 hours and found a significant rate of cell death compared to untreated cells (Mann–Whitney *U* test *p* < 0.001), as represented in Figure 4(a). When the same cells were exposed to PBM after 5-FU treatment, cell viability was significantly higher (Mann–Whitney *U* test *p* < 0.001).

Besides cell death, 5-FU also induced ROS production in human keratinocytes (Mann–Whitney *U* test *p* = 0.02), as assessed using the H_2_DCFDA dye, while PBM significantly lowered the level of intracellular ROS in both 5-FU treated and untreated cells (Mann–Whitney *U* test *p* = 0.048 and *p* = 0.015, respectively; Figure 4(b)).

We also assessed the expression levels of HMOX1 and SOD2, two enzymes playing a key role in the cellular response to oxidative stress, and consistently found that 5-FU upregulated both enzymes as expected, whereas PBM markedly decreased their expression (Mann–Whitney *U* test *p* = 0.03 for both genes), as shown in Figures 4(c) and 4(d). Similar to what was observed for ROS production, exposure of the cells to PBM resulted in a significant downregulation of HMOX1 and SOD2, even in cells treated with 5-FU (Mann–Whitney *U* test *p* = 0.03 for both genes, Figures 4(c) and 4(d)).

## 4. Discussion

Despite increasing evidence of its effectiveness in the treatment of OM and other inflammatory conditions, PBM still does not stand as a universally recognized and accepted therapy, essentially because of the variety of light sources and irradiation protocols employed in different trials in terms of wavelength (usually in the range of 600–1100 nm), irradiance, and fluence. Therefore, a major effort is needed to understand whether the different wavelengths exert specific biological effects and to establish the optimal parameters to be used to achieve the best therapeutic activity in each condition.

This work contributes to establishing the capacity of laser light to modulate oxidative stress, which is crucial in OM onset and progression. We report our clinical experience on cancer patients suffering from OM induced by either CT or RT. These patients were treated with a protocol optimized on the base of our previous clinical and experimental work [18, 24], using 970 nm laser light. Consistent with previous studies, this treatment was effective in improving all clinical parameters, starting from the fifth day of PBM. We then moved to assess the levels of ROS in the saliva of the same patients and observed a marked antioxidant activity exerted by each PBM session. However, this drop in ROS level was transient and not maintained during the 24 hours of posttreatment.

To better understand the cellular mechanism and the dynamics underlying this effect, with the ultimate goal of optimizing our clinical protocol, we wanted to assess the effect of multiple wavelengths of laser light on two different cell types, reasonably representing the major sources of ROS in OM, PMNs, and keratinocytes.

First, we evaluated the effect of red (660 nm) and near-infrared (970 nm) laser light on PMNs, either in resting conditions or upon stimulation with LPS and observed an opposite response. While the 970 nm light reduced ROS production in both conditions, the 660 nm light increased ROS levels in unstimulated PMNs and did not exert any effect in LPS-stimulated cells. Our result is also in accordance with the work by Cerdeira et al. [30], in which neutrophil irradiation with 660 nm laser light resulted in a significant increase in their respiratory burst, associated with intra- and extracellular superoxide radical production and improved fungicidal activity against *Candida albicans*. Our data add an important piece of evidence, showing that different wavelengths exert specific and even opposite effects on ROS production. While a definitive explanation for these differences is still missing, it might depend on the fact that intracellular chromophores, and in particular the cytochrome c oxidase, which is considered the main target of PBM, change their absorption spectra depending on their oxidation state [31].

This implies that PBM cannot be considered a single therapeutic entity and that the optimal wavelength can be chosen not only on the base of the depth of tissue penetration but also considering the biological activity exerted on the irradiated cells. This is particularly relevant considering that neutrophils of patients undergoing CT usually present a defective capacity in ROS generation, with reduced production of IL-1*β* and overall impaired antimicrobial function [32–34]. Thus, the use of a laser wavelength able to stimulate ROS production in PMNs may represent a powerful tool to boost their ability to respond to infections in immunocompromised patients.

To understand whether the same effects are also exerted on keratinocytes, we exploited genetically encoded redox biosensors to monitor ROS production in real time in response to PBM, this time also testing an additional wavelength (800 nm), which is being progressively used to reach inflamed tissues in depth, as this wavelength is poorly absorbed by water. We confirmed that the 660 nm laser light increases ROS production when applied either before or after an oxidative stimulus. More importantly, we found that the 970 nm laser light exerted a moderate antioxidant activity, whereas a striking reduction in the levels of ROS was detected in cells exposed to either the 800 nm laser light or the combination of the three different wavelengths.

Since this multiwavelength PBM protocol could represent a promising therapeutic tool to be introduced into the clinics, we wanted to confirm its actual capacity to reduce oxidative stress in keratinocytes treated with 5-FU treatment, partially mimicking the scenario of OM patients. We found that this combined PBM protocol reduced ROS levels and ROS-induced genes in both untreated and 5-FU-treated cells.

These results differ partially from those obtained using single wavelengths, in which PBM seems to decrease ROS levels in stressed or injured tissues, while increasing them in healthy ones [35]. For instance, Tatmatsu-Rocha et al. showed that the 904 nm laser light reduces oxidative stress markers in the wounded skin of diabetic mice, whereas it increased them in irradiated controls [36]. In primary cortical neurons, PBM at 810 nm increased ROS levels in basal conditions, but it reduced ROS when the neurons were treated with oxidant reagents [23]. The same wavelength was reported to lead to ROS increment in healthy murine embryonic fibroblast through the activation of nuclear factor kappa B (NF-*κ*B) [37]. Additional variability could depend on the timing at which ROS levels are assessed following PBM. While in healthy cells our combined PBM protocol did not induce any significant change in ROS levels during the first 5 minutes, as assessed using the fluorescent sensors, it was effective in reducing both ROS generation and ROS-induced gene expression after 18 hours, as determined by the H2DCFDA assay and real-time PCR.

These findings suggest that a combined multiwavelength irradiation protocol may be considered, to reach tissues located at different depths and exploit the different characteristics of single wavelengths, i.e., increasing ROS levels in neutrophils by the 660 nm laser light and reducing the oxidative stress in keratinocytes by the 800 and 970 nm light. Future studies using both cellular and animal models will further confirm the rationale and efficacy of this approach.

## 5. Conclusions

Overall, our study demonstrates that PBM exerts different effects on the redox state of both PMNs and keratinocytes, depending on the wavelength, and prompts the validation of a multiwavelength protocol in the clinical settings.

## Figures and Tables

**Table 1:** Selected baseline characteristics of the patients enrolled in the study.

Patient	Age	Gender	Malignancy	Anticancer therapy	VAS score at T0	CTC score at T0
1	58	M	Gastrointestinal	CT	2	2
2	58	F	Breast	CT	2	2
3	62	M	Head neck	CT and RT	8	2
4	92	F	Head neck	RT	8	2
5	74	M	Haematological	CT	7	3
6	56	M	Head neck	CT	5	2
7	65	M	Gastrointestinal	CT	6	3
8	44	M	Haematological	CT	2	2
9	69	M	Head neck	CT and RT	4	2
10	71	M	Head neck	RT	8	3

## Data Availability

All the data used to support the findings of this study are included in the article.
